# LCK expression is a potential biomarker for distinguishing primary central nervous system lymphoma from glioblastoma multiforme

**DOI:** 10.1002/2211-5463.12849

**Published:** 2020-04-13

**Authors:** Le Ge, Lixia Xu, Shan Lu, Hua Yan

**Affiliations:** ^1^ Tianjin Neurosurgical Institute Tianjin Key Laboratory of Cerebrovascular and Neurodegenerative Diseases Tianjin Huanhu Hospital China

**Keywords:** DLBCL, GBM, LCK, PCNSL

## Abstract

Glioblastoma multiforme (GBM) and primary central nervous system lymphoma (PCNSL) are both malignant cerebral tumors; however, their treatments are vastly different. Early and precise diagnosis is vital for subsequent adequate treatment to improve prognosis. Reliable biomarkers that can easily distinguish GBM and PCNSL are urgently needed. We evaluated the diagnostic potential of lymphocyte‐specific protein tyrosine kinase (LCK) as a biomarker in differentiating PCNSL from GBM using established computational approaches (Gene Expression Profiling Interactive Analysis, The Cancer Proteome Atlas, Tumor Immune Estimation Resource, GEO, Oncomine) and immunohistochemistry. The results showed that LCK was expressed at a high level in PCNSL patients but at a low level in GBM patients. Moreover, LCK expression positively correlated with the levels of infiltrating B cells in diffuse large B‐cell lymphoma (DLBCL) and GBM. Overall, bioinformatics analysis and immunohistochemistry revealed that LCK expression is a potential biomarker for distinguishing PCNSL from GBM.

## Graphical abstract

We evaluated the diagnostic potential of lymphocyte‐specific protein tyrosine kinase (LCK) as a biomarker in differentiating primary central nervous system lymphoma (PCNSL) from glioblastoma multiforme (GBM) using bioinformatics analysis (Gene Expression Profiling Interactive Analysis, The Cancer Proteome Atlas, Tumor Immune Estimation Resource, GEO, Oncomine) and immunohistochemistry. The results showed that LCK was expressed at a high level in PCNSL patients but at a low level in GBM patients, and positively correlated with the levels of infiltrating B cells.

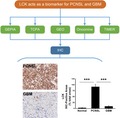

**Abbreviations**
DLBCL: Diffuse Large B‐cell Lymphoma
GBM: Glioblastoma multiforme
GEPIA: Gene Expression Profiling Interactive Analysis
LCK: Lymphocyte‐specific Protein Tyrosine Kinase
PCNSL: Primary Central Nervous System Lymphoma
TCPA: The Cancer Proteome Atlas
TIMER: Tumor Immune Estimation Resource

Glioblastoma multiforme (GBM) and primary central nervous system lymphoma (PCNSL) are malignant tumors of the central nervous system that have poor prognosis [1, 2]. The incidence of PCNSL has been increasing in the last few decades, while GBM is by far the most common primary glial tumor in adults. The diagnosis of PCNSL is often difficult because of its similarity to other brain tumors [2]. We aimed to study potential biomarkers for distinguishing PCNSL from GBM.

The current management strategies for the treatment of GBM and PCNSL are different, with the former being treated by a combination of maximal surgical resection with radiation treatment and concomitant and adjuvant chemotherapy with temozolomide, while patients with the latter condition undergo chemotherapy, targeted therapies, and whole brain radiation treatment [1]. The early and precise diagnosis of GBM and PCNSL is vital for the selection of a subsequent adequate treatment strategy to improve prognosis. However, PCNSL is diagnostically challenging [1, 2]. Various MR imaging features of PCNSL and GBM have been reported [3, 4, 5]; however, the most useful biomarkers for differentiating between PCNSL and GBM have not been evaluated. Thus, reliable biomarkers that can easily distinguish GBM and PCNSL are urgently needed.

Lymphocyte‐specific protein tyrosine kinase (LCK) is a member of the Src family of protein tyrosine kinases first identified in the 1980s [6, 7]. A multitude of studies have clarified the function of LCK in T lymphocytes, especially T‐cell receptor signaling [8, 9]. Additional studies have shown that LCK is not only expressed in T cells but also in other cell types and is involved in the downstream signaling of relative receptors [8]. Talab Fatima et al. reported that LCK plays an important role in mediating B‐cell receptor signaling in chronic lymphocytic leukemia cells [10]. In addition, although the expression of LCK was detected in different neural tissues, such as the hippocampus, cerebellum, and retina [11], its specific function is not yet clear.

In this study, we analyzed the expression of LCK in PCNSL and GBM through bioinformatics analysis and further confirmed its expression in patients by immunohistochemistry.

## Materials and methods

### Ethics statement

The study involving human tissue specimens was approved by the Ethics Committee of Tianjin Huanhu Hospital. All participators signed the informed consent form and were aware of the study details. This study conforms to the guidelines set by the Declaration of Helsinki.

### Clinical tissue specimens and immunohistochemistry

For immunohistochemical analysis, formalin‐fixed paraffin‐embedded samples from 20 PCNSL patients (median age 62 years; age range 51–68 years) and 20 systemic GBM patients (median age 63 years; age range 50–70 years) diagnosed at Tianjin Huanhu Hospital were used. The paraffin‐embedded brain tissues were sectioned into slides, and immunohistochemistry was performed on an YN‐05MY Automatic immunohistochemical staining system (YongNian, China) with an anti‐LCK antibody (Cat No. ab32149; Abcam, Cambridge, London, UK) and an anti‐phospho‐LCK (Tyr394) polyclonal antibody (Cat No. bs‐5406R; Bioss, Beijing, China). The percentage of LCK and phosphor LCK (Tyr394)‐positive cells were calculated subsequently.

### Oncomine database analysis

The Oncomine database (https://www.oncomine.org/resource/login.html) [12] was used to determine the expression of LCK in PCNSL and non‐CNS Diffuse Large B‐cell Lymphoma (DLBCL).

### The cancer proteome atlas (TCPA) database analysis

The Cancer Proteome Atlas (http://www.tcpaportal.org/tcpa) [13] was used to determine the protein expression level of LCK in PCNSL and GBM.

### Analysis in GEPIA

The online database Gene Expression Profiling Interactive Analysis (GEPIA) (http://gepia.cancer-pku.cn/index.html) [14] was used to determine the expression of LCK in DLBCL and GBM patients.

### Analysis in UALCAN

UALCAN (http://ualcan.path.uab.edu/) [15] is an interactive web portal for performing in‐depth analyses of TCGA gene expression data. UALCAN was used to clarify the expression and survival rates of LCK in DLBCL and GBM patients.

### Analysis in cBioPortal

The cBio cancer genomics portal (http://cbioportal.org) is an open‐access resource for the interactive exploration of multidimensional cancer genomics data sets, currently containing 225 cancer studies [16]. We used cBioPortal to analyze the biological interaction network of LCK derived from public pathway databases, with color‐coding and filter options based on the frequency of genomic alterations in each gene. Neighboring genes with alteration frequencies greater than 10% were included.

### Correlation analysis of LCK expression and immune infiltration level in DLBCL and GBM in TIMER

Tumor Immune Estimation Resource (TIMER; cistrome.shinyapps.io/timer) is devoted to comprehensively investigating the molecular characterization of tumor–immune interactions [17]. Here, we analyzed the correlation of LCK expression with immune cell infiltration in DLBCL and GBM. The immune cells included B cells, CD8+ T cells, DC cells, monocytes, neutrophils, NK cells, T cells, Treg cells, and so on.

### Statistical analysis

The expression results generated with established computational approaches are displayed as the fold change and *P* value. Survival curves were generated by the Kaplan–Meier method. The correlation of gene expression was evaluated by Spearman’s correlation and statistical significance, with *P* < 0.05 considered statistically significant.

## Results

### Determination of the expression level of LCK in DLBCL and GBM using UALCAN, GEPIA, and TCPA

**Fig. 1:**
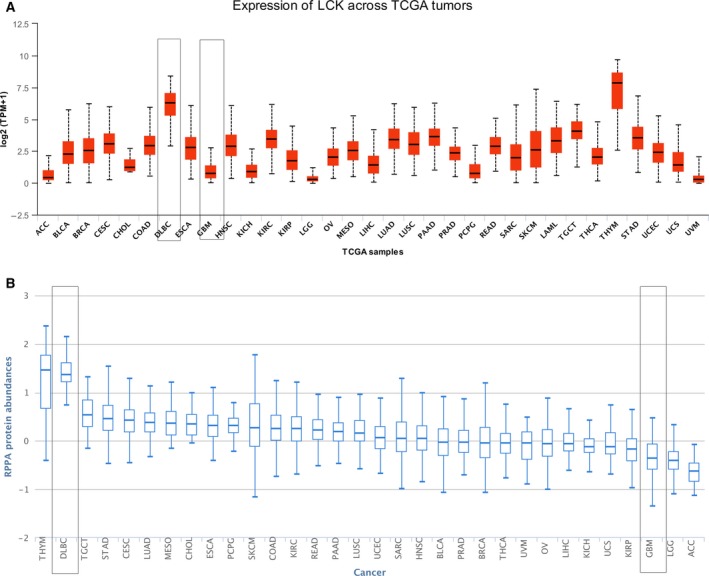
LCK expression in multiple tumor tissues. (A) LCK mRNA level determined using the UALCAN database. (B) LCK protein level determined with the TCPA database.

To determine differences in LCK expression in multiple tumor tissues, we applied established computational approaches (UALCAN, TCPA) to analyze the LCK mRNA and protein levels from The Cancer Genome Atlas (TCGA). The results from both UALCAN (Fig. 1A) and TCPA (Fig. 1B) showed that LCK expression was higher in thymoma and DLBCL. In addition, lower expression was observed in GBM, lower‐grade glioma (LGG), and adrenocortical carcinoma (ACC) (Fig. 1).

**Fig. 2:**
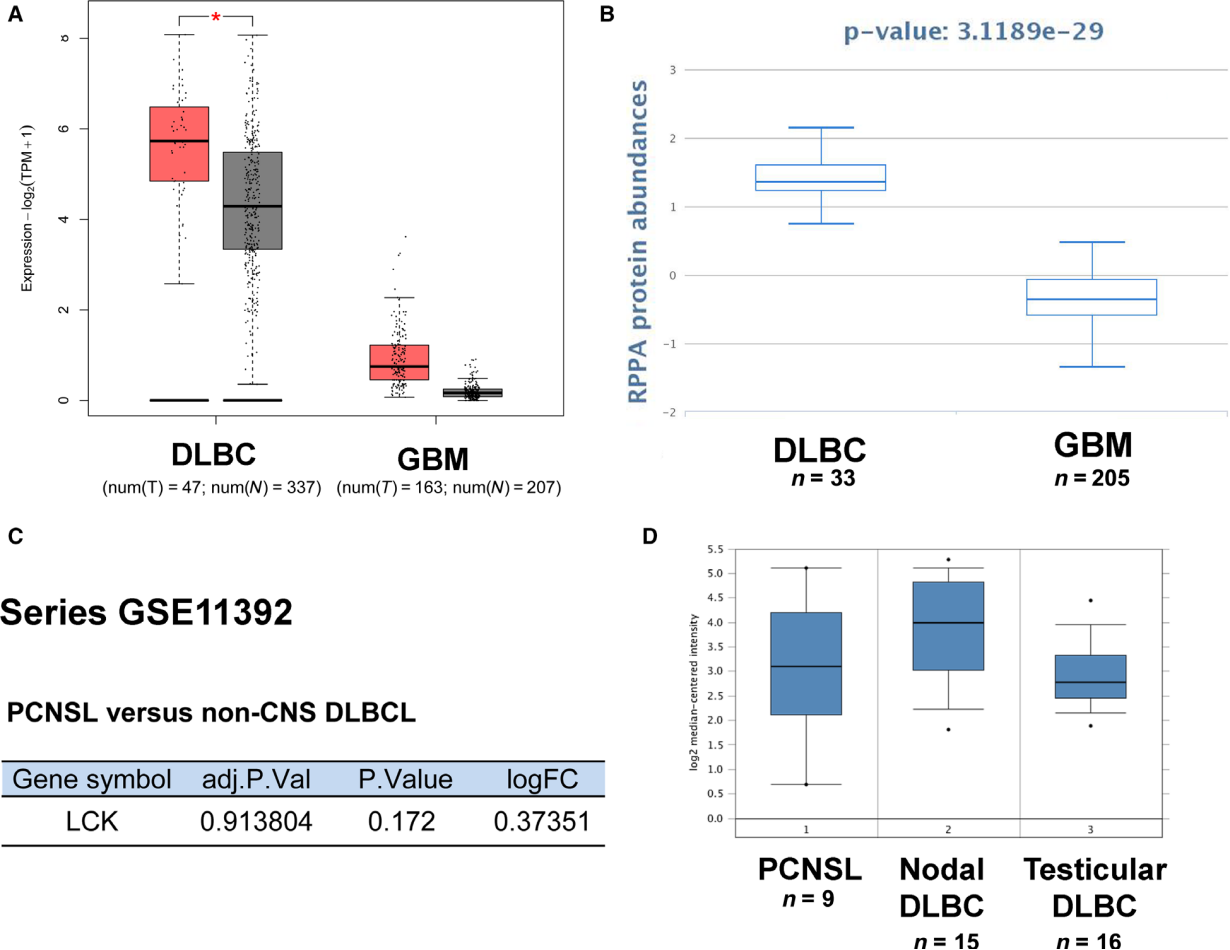
LCK expression in DLBLC and GBM using data mining. (A) LCK mRNA levels in DLBCL and GBM using GEPIA. (B) LCK protein levels in DLBCL and GBM using TCPA. (C‐D) No difference in LCK expression was observed between PCNSL and non‐CNS DLBCL using GEO (C) and Oncomine (D). *P < 0.01, one‐way ANOVA.

To further evaluate the diagnostic potential of LCK as a biomarker for differentiating CNS DLBCL from GBM, we examined the differential expression level between GBM and DLBCL using the GEPIA, TCPA, and GEO databases. The results from GEPIA (Fig. 2A) and TCPA (Fig. 2B) showed that the expression of LCK was markedly higher in the DLBCL group than in the GBM group. The GEO (series http://www.ncbi.nlm.nih.gov/geo/query/acc.cgi?acc=GSE11392) (Fig. 2C) and Oncomine (Fig. 2D) databases both showed that there were no differences in LCK expression between CNS DLBCL (PCNSL) and non‐CNS DLBCL. Taken together, these data mining analysis results showed that LCK may be used as a potential biomarker for distinguishing PCNSL from GBM.

### The expression and Tyr 394 phosphorylation level of LCK in PCNSL and GBM

**Fig. 3:**
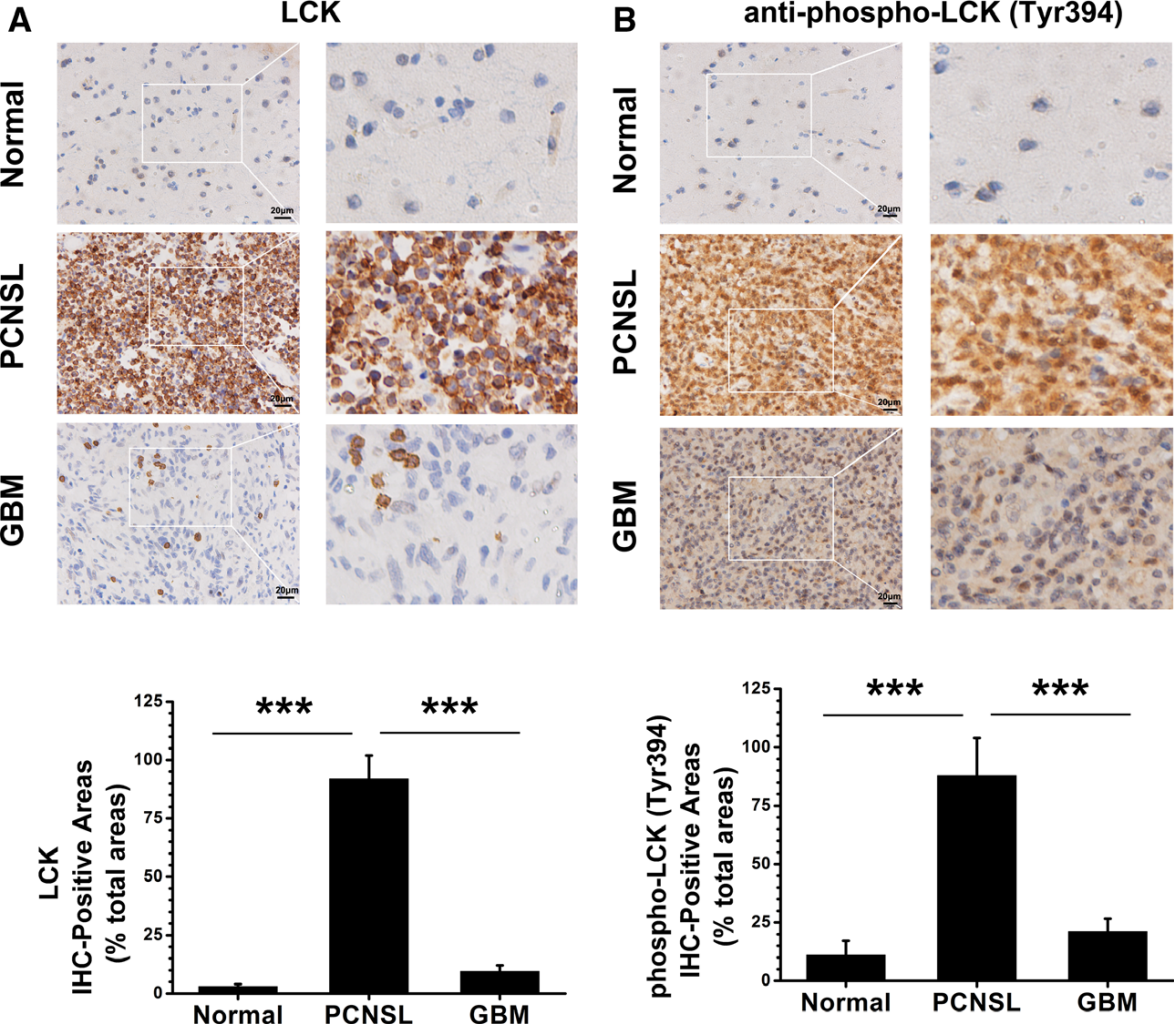
LCK is a potential biomarker for distinguishing PCNSL from GBM. Immunohistochemical analysis for LCK expression (A) and Tyr 394 phosphorylation level (B) in normal brain (*n* = 9), PCNSL (*n* = 20), and GBM (*n* = 20) tissues (scale bar = 20 μm). Statistical quantitative analysis listed below. The data are presented as the mean ± SD. ***P < 0.001, one‐way ANOVA.

Primary central nervous system lymphoma is a unique and aggressive subtype of extranodal lymphoma and is always correlated with poor prognosis [1]. The diagnosis of PCNSL is often difficult because of its similarity to other brain tumors [8]. We aimed to study potential biomarkers for distinguishing PCNSL from GBM. Immunohistochemistry analysis was used to detect the expression of LCK in PCNSL and GBM (Fig. 3A, Table 1). We found that the expression level of LCK in the PCNSL group was significantly higher than that in the GBM group (Fig. 3A). Table 1 shows that in the PCNSL group, the LCK expression was 50% (10/20) strongly expressed (+++, >90%), 20% (4/20) moderately expressed (++, 30%‐90%), and 30% (6/20) weakly expressed (+, 10–30%), with no negative expression (−, <10%). In the GBM group, there was no strong positive expression, with only 20% (4/20) moderate expression, 45% (9/20) weak expression, and 35% (7/20) negative expression. Because phosphorylation of Tyr‐394 activates LCK, we further analyzed the activity of LCK in PCNSL and GBM by using an anti‐phosphotyrosine 394 antibody (Fig. 3B, Table 1). Immunohistochemistry data showed that the Tyr 394 phosphorylation level of LCK in the PCNSL group was significantly higher than that in the GBM group, which was similar to the expression level of LCK (Fig. 3B, Table 1). These immunohistochemistry analysis results confirmed that LCK can be used as a potential biomarker for distinguishing PCNSL from GBM.

**Table 1:** Expression and Tyr 394 phosphorylation level of LCK in PCNSL and GBM.

	Negative− (< 10%)	Weak+ (10–30%)	Positive ++ (30–90%)	Strong+++ (> 90%)
LCK				
Normal	55.6% (5/9)	33.3% (3/9)	11.1% (1/9)	0% (0/9)
PCNSL	0% (0/20)	30% (6/20)	20% (4/20)	50% (10/20)
GBM	35% (7/20)	45% (9/20)	20% (4/20)	0% (0/20)
phospho‐LCK (Tyr394)				
Normal	44.4% (4/9)	44.4% (4/9)	11.1% (1/9)	0% (0/9)
PCNSL	0% (0/20)	25% (5/20)	30% (6/20)	45% (9/20)
GBM	30% (6/20)	45% (9/20)	25% (5/20)	0% (0/20)

### Prognosis and biological interaction network of LCK in PCNSL and GBM

**Fig. 4:**
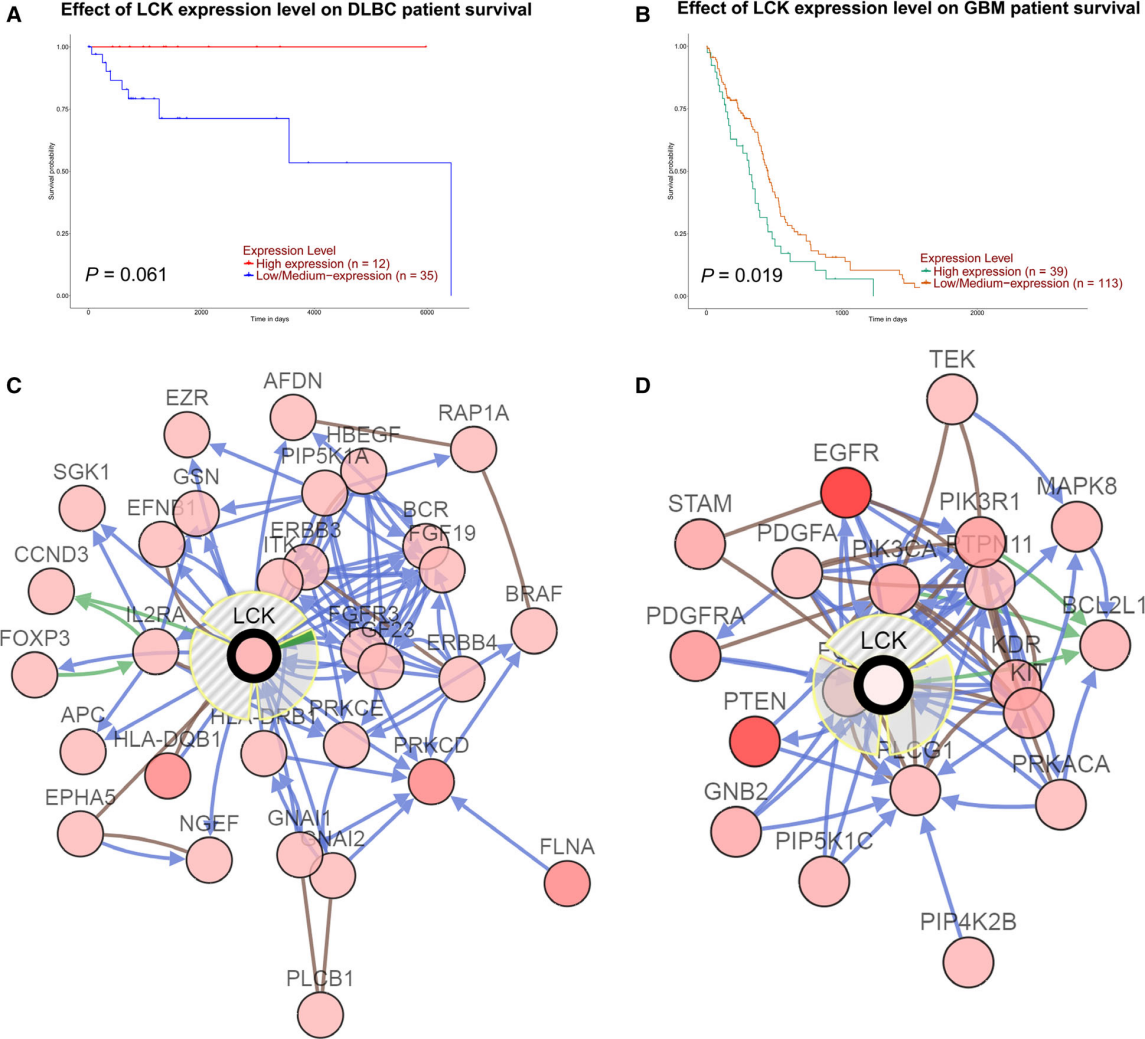
Prognosis and biological interaction network of LCK in PCNSL and GBM. (A‐B) Effect of LCK expression level on DLBCL (A) and GBM (B) survival using UALCAN. (C‐D) Network view of the LCK neighborhood in PCNSL (C) and GBM (B).

To evaluate the prognostic significance of LCK expression, we analyzed the influence of LCK expression on survival rates with UALCAN (Fig. 4A,B). We found that lower LCK expression was associated with poor survival in DLBCL (*P* = 0.061, *n* = 47, Fig. 4A) and GBM (*P* = 0.019, *n* = 152, Fig. 4B). To determine the biological interaction network of LCK in PCNSL and GBM, we used the tab network in cBioPortal to show LCK‐neighboring genes that were altered at frequencies > 10% in PCNSL and GBM (Fig. 4C,D). The neighboring genes of LCK with the most frequent alterations in GBM were EGFR (59%) and PTEN (42.1%) and that in PCNSL were FLNA (20%) and HLA‐DQB1 (20%).

### LCK expression was correlated with immune infiltration levels in DLBCL and GBM

**Fig. 5:**
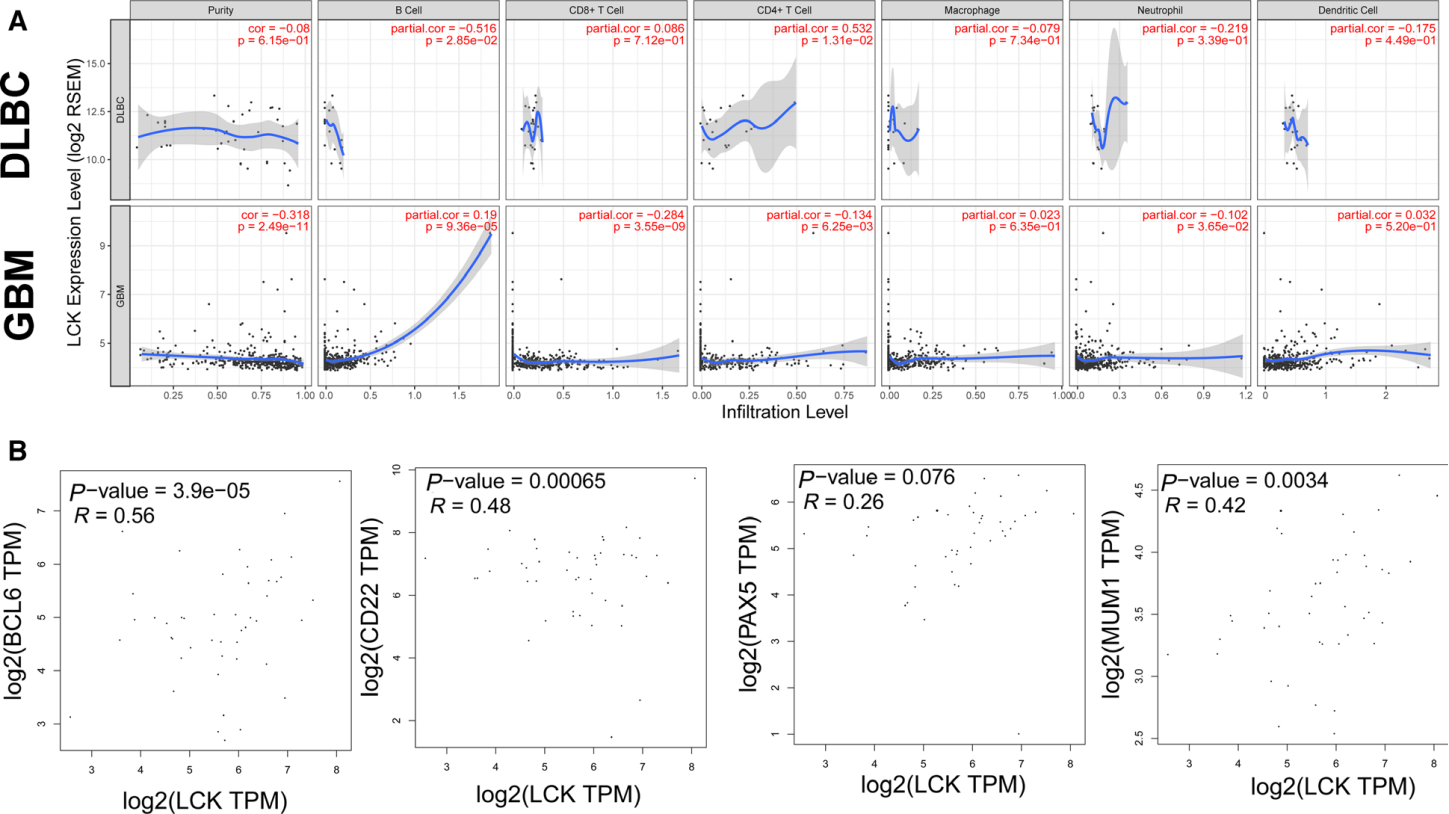
Correlation of LCK expression with immune infiltration level in DLBCL and GBM. (A) LCK expression is significantly positively correlated with the infiltrating levels of B cells in GBM. (B) Correlation analysis between LCK and related genes and markers of B cells in GEPIA..

Tumor‐infiltrating lymphocytes are closely related to the prognosis of cancers. Therefore, the correlation of LCK expression and immune infiltration levels in GBM was determined in TIMER (Fig. 5A). The data from the ‘gene’ module of TIMER showed that LCK mRNA expression had a significant negative correlation with tumor purity in GBM, but not in DLBCL (GBM, *r *= −0.318, *P* = 2.49E‐11; DLBCL, *r* = −0.08, *P* = 6.15E‐1). Moreover, LCK expression positively correlated with the B‐cell infiltration level in GBM (*r* = 0.19, *P* = 9.36E‐5) (Fig. 5A). In DLBCL, we further assessed the correlation between the LCK expression level and B‐cell‐correlated biomarkers with GEPIA (Fig. 5B). The results showed a positive correlation between LCK expression and BCL6 (*r* = 0.56, *P* = 3.9E‐5), CD23 (*r* = 0.48, *P* = 0.00065), MUM1 (*r* = 0.42, *P* = 0.0034), and PAX5 (*r* = 0.26, *P* = 0.076) expression, which are very common markers for the pathological identification of DLBCL. According to the above results, we concluded that LCK expression correlated with B‐cell immune infiltration levels in DLBCL and GBM.

## Discussion

Lymphocyte‐specific protein tyrosine kinase, a nonreceptor Src family kinase, plays a crucial role in various cellular processes, such as cell cycle control, cell adhesion, migration, proliferation, and differentiation. In addition to the important role of LCK in the function of T lymphocytes [8, 9], there is increasing evidence that LCK is also widely expressed in brain [11] and tumor cells [18], and it actively participates in signal transduction processes such as cell proliferation, survival, and memory. Therefore, LCK has emerged as a novel druggable target molecule for the treatment of cancer and neuronal diseases [19, 20]. Consistent with this notion, it has been reported that LCK‐targeted inhibitors can regulate human glioma cell migration, tumor growth, and stemness gene expression [18]. Similarly, LCK is involved in the fractionated radiation‐induced expansion of the glioma‐initiating cell population and decreased cellular sensitivity to anticancer treatments [21]. LCK appears to be expressed in primary glioma cells but not in the paired glioma stem cells from the same human GBM tumors, which indicates that LCK activity is not necessary for the maintenance of glioma stem cells [22]. However, the expression level and prognosis of LCK in GBM and DLBCL tissues have not been elucidated. Our results showed that LCK expression was the highest in thymoma and DLBCL, whereas it was lower in GBM, LGG, and ACC (Fig. 1), and the lower LCK expression in DLBCL and GBM predicted poor survival. LCK expression was correlated with immune infiltration levels in DLBCL and GBM. These results indicated that LCK can serve as an independent novel prognostic gene in GBM and DLBCL.

Another important highlight of our study is the potential of LCK expression for distinguishing PCNSL from GBM. PCNSL is a distinct subtype of extranodal DLBCL with aggressive ability and poor prognosis [1]. The diagnosis of PCNSL is often difficult because of its similarity to other brain tumors. Various MR imaging features of PCNSL and GBM have been reported [12, 13]; however, the most useful biomarkers for the differentiation between PCNSL and GBM have not been evaluated. Our results showed that the expression level of LCK in the PCNSL group was significantly higher than that in the GBM group, which confirmed that LCK can be used as a potential biomarker for distinguishing PCNSL from GBM.

In summary, we found that LCK can serve as an independent novel prognostic gene in GBM and DLBCL. In addition to its potential usefulness as a clinical biomarker, LCK is expected to be a biomarker for distinguishing PCNSL from GBM.

## Conflict of interest

The authors declare that there are no conflicts of interest.

## Author contributions

HY and LG designed the study. LG and LX performed the IHC staining and data mining. SL performed the data analysis. LG, LX, and SL contributed equally to this study.
